# The Rise of Mpox in a Post-Smallpox World

**DOI:** 10.3201/eid3101.241230

**Published:** 2025-01

**Authors:** Jennifer H. McQuiston, Andrea McCollum, Athalia Christie, Fernando Torres, Jonathan Mermin, Daniel B. Jernigan, Christina L. Hutson

**Affiliations:** Centers for Disease Control and Prevention, Atlanta, Georgia, USA

**Keywords:** mpox, monkeypox virus, smallpox, outbreak, orthopox vaccine, vaccines, viruses

## Abstract

Reports of mpox are rising in Africa where the disease is endemic and in new countries where the disease has not been previously seen. The 2022 global outbreak of clade II mpox and an ongoing outbreak of the more lethal clade I mpox highlight the pandemic potential for monkeypox virus. Waning population immunity after the cessation of routine immunization for smallpox plays a key role in the changing epidemiologic patterns of mpox. Sustained human-to-human transmission of mpox is occurring widely in the context of insufficient population immunity, fueling genetic mutations that affect the accuracy of some diagnostic tests and that could lead to changing virulence. Additional research should address complex challenges for control of mpox, including improved diagnostics and medical countermeasures. The availability of vaccines should be expanded not only for outbreak response but also for broader routine use for persons in mpox-endemic countries.

Monkeypox virus (MPXV), represented by 2 virus clades and several subclades with unique genetic, pathogenic, and geographic characteristics (*1*), is increasingly in the scientific and public spotlight. Although clade II MPXV has historically led to a case-fatality rate of 3%–4%, clade I MPXV has been shown to cause severe illness and death in a higher proportion of patients; case-fatality rates among unvaccinated persons are up to 10%–11% (*1*). In 2022, clade IIb MPXV caused a large global outbreak that predominantly spread via sexual contact among gay, bisexual, and other men who have sex with men (MSM) (*2*). Since 2022, that outbreak has continued at low levels; ≈100,000 cases have been reported through August 2024 (*3*). More recently, since 2023 an outbreak of clade I MPXV in the Democratic Republic of the Congo (DRC) has resulted in tens of thousands of suspected cases and subsequent spread to neighboring countries (*4*,*5*). That outbreak is affecting persons across a range of ages and genders; complex transmission drivers are still being investigated (*4*), and household spread and sexual transmission, including within heterosexual networks, have been reported (*4*).

Even before the recent outbreaks, scientists have been warning of steady increases in both clade I and clade II mpox in mpox-enzootic countries in Africa for years. From Cameroon, Central African Republic, DRC, Nigeria, and Republic of Congo, ≈1,620 mpox cases were reported during the 45-year period 1970–2015 (mean 36 cases/year) (*6*). In contrast, 25,488 cases were reported from those countries during the 6-year period 2016–2021 (mean 4,248 cases/year) (*6*). In Nigeria, reports of clade II MPXV infection began to increase in 2017, before the virus eventually spread around the world in 2022 (*7*,*8*). The outbreak of clade I MPXV in DRC is showing signs of following a similar trajectory with recent regional spread (*5*,*9*). As of December 2024, cases in travelers have been reported from numerous countries, including the United States, Canada, and the United Kingdom (*10*–*12*). The DRC clade I mpox outbreak poses a serious risk for further global spread, causing the World Health Organization (WHO) to declare a Public Health Emergency of International Concern on August 14, 2024 (*13*).

The pandemic potential of mpox has long been overshadowed by the historical focus on smallpox (caused by variola virus). The 2 orthopoxviruses are genetically related and result in similar clinical manifestations (albeit with differing degrees of severity). They are also prevented by the same vaccinia virus–based vaccines. Smallpox eradication, which was achieved in 1979, was made possible in part because humans were the only host, enabling an intensive eradication campaign (*14*,*15*). In contrast, mpox occupies a more complex niche as a zoonotic disease with secondary communicable spread to humans.

Several factors may contribute to the emergence of mpox as a post-smallpox threat. Increased human exposure to wildlife reservoirs is hypothesized to contribute to the rise in mpox cases. The exposures are multifactorial, from an increase in the absolute number of persons living in the mpox-endemic countries of western and central Africa, to habitat encroachment, to a reliance on wild animal protein in food-scarce forested regions (*16*). More recently, it has become clear that MPXV of either clade is efficiently spread from human to human via sexual contact; rapid geographic spread is driven by transboundary movement of persons via migration and travel (*17*,*18*). That pattern was seen with clade IIb MPXV in 2022 and with the expansion of the DRC clade I outbreak eastward to neighboring countries in 2024 (*17*,*18*). In addition to factors driving increased human exposure, improved surveillance contributes to increased detection and reporting, although surveillance systems in DRC during the early 2000s showed rising incidence even during relatively stable reporting periods (*1*,*21*).

Another factor influencing the rise of mpox is diminishing population immunity to orthopoxviruses (*19*). Routine immunization with vaccinia virus–derived vaccines stopped worldwide after smallpox eradication was announced by the WHO on December 9, 1979 (*14*,*15*,*21*). Residual immunity to orthopoxviruses resulting from smallpox vaccinations has been protecting humans against mpox for decades, but the effects of diminishing immunity to MPXV have long been predicted (*22*). Past vaccine recipients now make up a minority of the world’s population. Currently, the only persons with a history of vaccination are those who received a routine smallpox vaccination (most often delivered as a childhood vaccine before the eradication of smallpox) and those who were immunized as part of military service, because of occupational risks, or in response to the recent clade II mpox outbreak. The loss of population immunity in MPXV-enzootic parts of the world increases the chance that spillover infections may occur and also increases the risk for subsequent sustained person-to-person spread of mpox.

During the 1970s–1980s, mpox was predominantly a disease of children; median patient age was 4–5 years (*19*). However, during 2010–2019, the median age of persons with mpox increased to 21 years (*19*), coincident with a loss of population immunity across most age groups. It has been estimated that for every 1% decrease in herd immunity, mpox incidence increases by 0.13 cases/100,000 population (*23*). Modeling suggests that as population immunity levels fall, the estimated basic reproduction number (the anticipated number of secondary infections for each primary infection in a fully susceptible population) and epidemic potential for clade I MPXV rises; at 40% immunity, clade I MPXV is estimated to have a basic reproduction number of 2.1 (range 1.5–2.7) (*24*). As a recent tangible example of the pandemic potential for MPXV, in 2016, only ≈10% of the population in Nigeria had previously received a smallpox vaccine, and serologic studies suggested general population immunity levels of 2.6% (*7*). Retrospective analysis of genomic data from MPXV in Nigeria now estimates that clade IIb MPXV probably began circulating from person to person in Nigeria around 2016 (*8*). Most parts of the world currently have orthopoxvirus population immunity estimates <20%, although levels of immunity may be higher for certain groups (i.e., gay, bisexual, or other MSM with behavioral factors associated with risk for mpox exposure) because of infection with clade IIb or recent vaccination (*25*).

Mpox in the modern era is also influenced by new complexities, including underlying medical conditions. Risk for severe or fatal illness is increased among patients with mpox and substantial immunocompromise, including HIV-associated immunosuppression or other conditions or medications that impair the immune response (*26*). Smallpox eradication predated recognition of the global HIV pandemic, but an estimated 26 million persons live with HIV in Africa, and HIV remains a leading cause of death in some parts of the continent (*27*). In addition, humans are now more mobile than they were during the smallpox era. In 2022, the clade IIb mpox outbreak spread rapidly around the globe in just a few months. It was initially dispersed by travelers who extended regional spread via sexual contact (*17*). The clade IIb outbreak demonstrated the pandemic potential of mpox but resulted in relatively few deaths (US case-fatality rate <0.2%) (*2*). In contrast, clade I MPXV is considered more virulent, leading to death for nearly 5% of persons with suspected cases in DRC (*4*). Still, a global outbreak of clade I mpox would probably result in lower mortality rates than have been observed to date in DRC. Recent studies have demonstrated improved survival rates (case-fatality rates 1.4%–1.7%) among persons with access to basic medical care and nutritional support (*28*,*29*); mortality rates are expected to be lower in countries with strong healthcare systems, such as the United States.

The recent rise of mpox introduces another factor for concern: the risk for new genomic mutations. Such mutations, which would be expected to accumulate as a result of extensive person-to-person spread, could increase MPXV virulence or decrease the effectiveness of diagnostics or medical countermeasures. Although DNA viruses mutate at much slower rates than RNA viruses (e.g., influenza or SARS-CoV-2 virus), they do change with time. There is already evidence in a subset of clade I MPXV specimens of a large deletion in the MPXV genome affecting the specificity of 1 diagnostic test (*18*). There is also evidence of other genetic changes in the same clade I virus specimens from eastern DRC, where sustained person-to-person transmission is occurring through sexual contact (*18*). Those changes do not seem to enhance virulence, and the large deletion is actually hypothesized to potentially decrease virulence (*30*). However, future genomic changes driven by ongoing communicable spread could alter the virus in a way that increases virulence.

To control outbreaks and protect against a potential future pandemic, human-to-human spread of MPXV must be minimized, which should include reducing clade I and clade IIb MPXV transmission through sexual contact, as well as preventing and controlling outbreaks. The WHO developed a recent strategic framework for prevention and control of mpox, with a goal of eliminating human-to-human transmission (*31*). Given the evidence that both MPXV clades can spread efficiently via sexual contact and the disproportionate effect of clade IIb mpox among gay, bisexual, and other MSM who have been historically marginalized and stigmatized, a syndemic approach to response and prevention is advised. That approach includes incorporating mpox prevention strategies for at-risk populations into routine sexual healthcare, making efforts to promote community engagement and health equity, and investing in sexual health programs that address multiple infectious diseases, including mpox (*32*).

Although population immunity is not the only factor influencing the spread of mpox in the modern era, it is a variable with a proven intervention the world has at the ready: vaccine. Unlike older smallpox vaccines, which posed a risk for complications in persons with immune compromise, a newer third-generation nonreplicating vaccine (JYNNEOS/MVA [modified vaccinia Ankara]; Bavarian Nordic, https://www.bavarian-nordic.com) is safe for use in immunocompromised persons (*33*). It has also demonstrated high efficacy against clade IIb (*34*,*35*). It is licensed for use in adults in many countries around the world but has not been extensively used to date in any countries where MPXV is enzootic. A first step is overcoming regulatory hurdles preventing routine use in MPXV-enzootic countries. Before September 2024, the lack of WHO prequalification of JYNNEOS/MVA complicated timely country approvals and created challenges for procurement by United Nations agencies. The September 13, 2024, announcement of the inclusion of JYNNEOS/MVA on the WHO vaccine prequalification list opened new opportunities for expanded vaccine procurement and easier approval pathways in MPXV-enzootic countries (*36*). Multiple donors have pledged support to donate or purchase vaccine for DRC, and some vaccine doses have begun arriving in the country (*37*,*38*). However, managing the financial and programmatic logistics of a vaccination campaign presents additional challenges in resource-limited settings. The vaccine is costly, has specific cold chain and handling requirements, and needs 2 doses spaced a month apart for full protection. Despite those challenges, with strategic use, the vaccine could protect against a global pandemic at its source, instead of merely being stockpiled for use in non–mpox-endemic countries where few cases may occur.

Moving forward, control of mpox is best supported through vaccination. In non–mpox-endemic countries with new or ongoing outbreaks, a risk-based approach for vaccine recommendations is key; epidemiologic studies will help define those at increased risk. In the United States, vaccination is currently recommended for laboratory workers who may encounter virus during laboratory procedures and for persons with certain sexual behavioral risk factors for exposure to clade IIb MPXV (*39*). In Central Africa, where the clade I MPXV outbreak is spreading and where vaccines may currently be limited in quantity, targeted vaccination efforts are needed, which might include vaccinating children, healthcare workers, persons at risk for zoonotic exposure, and those at increased risk for person-to-person spread because of specific behaviors or occupations. However, for long-term control of mpox and prevention of future outbreaks, broader immunization strategies for persons at increased risk for mpox infection should be considered, which, depending on vaccine availability, might include more expansive preexposure vaccination recommendations for persons living in MPXV-enzootic areas or countries.

In addition to expanded vaccine access and use, new global investments in MPXV research are critical. The United States had invested 2 decades in smallpox preparedness planning before the recent rise of mpox (*40*), which resulted in reliable diagnostics, a licensed vaccine, and investigational medical countermeasures being available from the start of the 2022 clade IIb mpox outbreak. However, research gaps have also been identified, including limited therapeutic options for severely immunocompromised persons and rare but concerning MPXV genomic changes conferring resistance to the antiviral therapeutic tecovirimat (*41*). Furthermore, recent findings from a clinical trial in DRC did not show a clinical benefit among clade I mpox patients treated with tecovirimat (*29*), highlighting the value of additional studies and new antiviral drug development pathways. During the clade IIb mpox outbreak, there were challenges with vaccine uptake and early decisions around distribution of limited vaccine stocks. In resource-limited countries, lack of a temperature-stable point-of-care diagnostic test appropriate for worldwide use delayed accurate diagnoses. Continued research into new diagnostics and vaccines is urgently needed, particularly single-dose vaccines with temperature-stable handling requirements.

The response to recent mpox outbreaks has clearly benefitted from programmatic efforts for smallpox biothreat preparedness. However, smallpox preparedness focused on scientific solutions to theoretical risks. Mpox is a real and current threat to global health security, and meaningful future control will require a complex partnership between governments, public health experts, virologists, chemists, and funders. It will also require reimagining how orthopox vaccine is shared and used around the world. We now need to develop the programmatic infrastructure to address mpox as a current pandemic threat, independent of the shadow of smallpox.
